# Multifaceted Function of Myosin-18, an Unconventional Class of the Myosin Superfamily

**DOI:** 10.3389/fcell.2021.632445

**Published:** 2021-02-09

**Authors:** Zhaohui Ouyang, Shuangshuang Zhao, Su Yao, Jing Wang, Yanqin Cui, Ke Wei, Yaming Jiu

**Affiliations:** ^1^Institute for Regenerative Medicine, Shanghai East Hospital, Shanghai Key Laboratory of Signaling and Disease Research, Frontier Science Center for Stem Cell Research, Ministry of Education of China, School of Life Sciences and Technology, Tongji University, Shanghai, China; ^2^The Joint Program in Infection and Immunity, Guangzhou Women and Children’s Medical Center, Guangzhou Medical University, Guangzhou, China; ^3^Institut Pasteur of Shanghai, Chinese Academy of Sciences, Shanghai, China; ^4^Unit of Cell Biology and Imaging Study of Pathogen Host Interaction, The Center for Microbes, Development and Health, Key Laboratory of Molecular Virology and Immunology, Institut Pasteur of Shanghai, Chinese Academy of Sciences, Shanghai, China; ^5^University of Chinese Academy of Sciences, Beijing, China

**Keywords:** myosin-18A, myosin-18B, biomechanics, muscle development, cancer

## Abstract

Myosin is a diverse superfamily of motor proteins responsible for actin-based motility and contractility in eukaryotic cells. Myosin-18 family, including myosin-18A and myosin-18B, belongs to an unconventional class of myosin, which lacks ATPase motor activity, and the investigations on their functions and molecular mechanisms in vertebrate development and diseases have just been initiated in recent years. Myosin-18A is ubiquitously expressed in mammalian cells, whereas myosin-18B shows strong enrichment in striated muscles. Myosin-18 family is important for cell motility, sarcomere formation, and mechanosensing, mostly by interacting with other cytoskeletal proteins and cellular apparatus. Myosin-18A participates in several intracellular transport processes, such as Golgi trafficking, and has multiple roles in focal adhesions, stress fibers, and lamellipodia formation. Myosin-18B, on the other hand, participates in actomyosin alignment and sarcomere assembly, thus relating to cell migration and muscle contractility. Mutations of either *Myo18a* or *Myo18b* cause cardiac developmental defects in mouse, emphasizing their crucial role in muscle development and cardiac diseases. In this review, we revisit the discovery history of myosin-18s and summarize the evolving understanding of the molecular functions of myosin-18A and myosin-18B, with an emphasis on their separate yet closely related functions in cell motility and contraction. Moreover, we discuss the diseases tightly associated with myosin-18s, especially cardiovascular defects and cancer, as well as highlight the unanswered questions and potential future research perspectives on myosin-18s.

## Introduction

Myosins are a large superfamily of proteins that are responsible for providing motility of various components in the cells and motility of cells, tissues, and organs. Following the discovery of the major motor proteins in striated muscles, namely, the class II myosins, more and more different subfamilies of myosins have been identified in animals. All myosins contain a motor domain, which in most cases is an ATPase, and can hydrolyze an ATP to create conformational changes, underlining the motor function of the myosins. However, not all myosin motors possess the ATPase-driven motor activity, exemplified by the recently discovered myosin-18 family consisting of myosin-18A and myosin-18B. This unconventional family of myosin has been found to be associated with multiple cellular processes and implicated in a wide range of diseases, including cancer and myopathy (Taft et al., 2014).

### Gene Structure and Expression of Myosin-18s

In 2000, the first member of myosin-18s, myosin-18A, was identified in bone marrow stromal cells through a differential display screen (Furusawa et al., 2000). MYO18A, the founding member of the class XVIII myosins, was initially named as MysPDZ (myosin-containing PDZ domain) (Furusawa et al., 2000) and later renamed myosin-18A because of discovery of other closely related genes. The *MYO18B* gene was annotated in 2002 (Nishioka et al., 2002) and was found to be present in vertebrates only (Salamon et al., 2003). Myosin-18A was found in a much broader range of animals, implying that myosin-18B could be derived from myosin-18A *via* a duplication event during evolution (Salamon et al., 2003).

**FIGURE 1:**
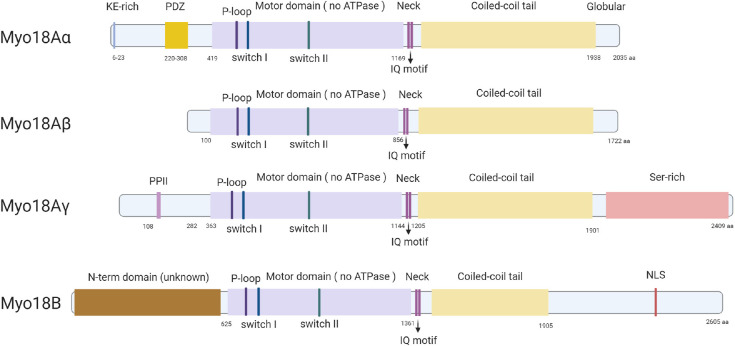
Protein structures of all known isoforms of myosin-18A and myosin-18B. All known isoforms of myosin-18A and myosin-18B contain a central motor domain and a coiled-coil tail domain linked by a short neck domain with two IQ motifs, whereas different isoforms are different in their distinct N-terminal and C-terminal extensions. Myosin-18Aα has a KE-rich region and a PDZ domain in its N-terminal extension, and myosin-18Aγ contains a proline-rich PPII domain in the N-terminal and a long serine-rich C-terminal. Myosin-18B has both long N- and C-terminals, and an NLS domain in the C-terminal extension is the only domain with a predicted function.

In mouse, *MYO18A* is located on chromosome 11 and encodes for three major splice isoforms, a long myosin-18Aα, a short myosin-18Aβ, and myosin-18Aγ, which is specifically expressed in striated muscle (Horsthemke et al., 2019; Figure 1). Myosin-18Aα consists of 2,035-amino-acid residues (∼230 kDa), myosin-18Aβ has 1,719-amino-acid residues (∼196 kDa), and myosin-18Aγ has 2,409-amino-acid residues (267 kDa), respectively. The human *MYO18A* gene is on chromosome 17, and human myosin-18Aα comprises 2,054-amino-acid residues (∼233 kDa), and myosin-18Aβ has 1,723-amino-acid residues (∼196 kDa). Mouse *M*yo*18B* is located on chromosome 5 and produces myosin-18B with 2,605-amino-acid residues (∼288 kDa), whereas human *MYO18B* gene is on chromosome 22, and it encoded a 2567-amino-acid residues (∼285 kDa) myosin-18B. The alternative splicing of myosin-18B was poorly understood; it is possible that various isoforms of myosin-18B similar to myosin-18A exist.

The central region of all isoforms of myosin-18A and myosin-18B contains a motor domain, followed by a short light chain–binding domain and a coiled-coil tail domain. These are the core features of most myosins and exhibit the highest similarity with the conventional muscle myosin-2 (Taft and Latham, 2020; Figure 1). The fundamental function of the motor domain of classic myosins is the ATPase activity, which causes conformational changes after hydrolyzing an ATP, thus providing traction between myosin and actin filaments and ultimately movement in cells and tissues (Preller and Manstein, 2013). And the coiled-coil domain allows dimerization of myosins (Preller and Manstein, 2013). Importantly, a few key amino acid residues highly conserved in the motor domain of classic myosins are mutated in both members of myosin-18 family (Taft and Latham, 2020). Particularly, two highly conserved serine residues known for facilitating efficient catalysis of the ATPase are mutated to alanine and threonine in both myosin-18A and myosin-18B (Taft and Latham, 2020). This finding prompted researchers to examine whether myosin-18s have motor functions. This important biochemical activity is thoroughly examined in myosin-18A from *Drosophila* (Guzik-Lendrum et al., 2011), mouse (Guzik-Lendrum et al., 2013), and human (Taft et al., 2013). No ATPase activity was found in any of the isoforms of myosin-18A, while actin binding was evident in all forms (Guzik-Lendrum et al., 2011, 2013; Taft et al., 2013), suggesting myosin-18A lacks motor activity and thus is an unconventional myosin. Although such detailed biochemical characterization of myosin-18B was not available, it does have the same mutations in the core motor domain as myosin-18A (Figure 1), and subsequent research assumes myosin-18B also lacks motor activity (Jiu et al., 2019). Following the motor domain, both myosin-18s have a short light chain–binding domain, which binds to the essential light chain (ELC) and regulatory light chain (RLC) in conventional class II muscle myosins (Preller and Manstein, 2013). The light chain–binding domain of all isoforms of myosin-18A and myosin-18B has two IQ motifs, which are known for interacting with calmodulin (CaM) or CaM-like light chains (Odronitz and Kollmar, 2007). Direct interaction of myosin-18A with ELC and RLC was experimentally confirmed (Guzik-Lendrum et al., 2013), whereas myosin-18B is expected to be capable of similar interactions due to sequence similarity. The coiled-coil domain is important for dimerization and incorporation into non-muscle myosin 2 (NM2) filaments. Both myosin-18A and myosin-18B do exhibit such activities (Ajima et al., 2008; Billington et al., 2015; Jiu et al., 2019), which is not different from conventional myosins. Flanking the generic motor domain and coiled-coil domain of myosin-18A and myosin-18B are long N-terminal and C-terminal domains, which are distinctive features of myosin-18 family proteins. The N-terminal domain of myosin-18Aα contains a KE motif rich in lysine and glutamate residues, as well as a PDZ domain, which was the reason myosin-18A was initially named as MysPDZ for “myosin-containing PDZ domain” (Furusawa et al., 2000). While myosin-18Aβ lacks an N-terminal domain, myosin-18Aγ has a long N-terminal domain with a unique sequence of unknown function except a short polyproline II (PPII) helix (Horsthemke et al., 2019; Figure 1). Myosin-18B also has large N-terminal and C-terminal domains. These domains are less studied, and they exhibit little similarity to known sequences, except a putative nuclear localization sequence (NLS) in its C-terminal domain (Salamon et al., 2003).

Myosin-18Aα is ubiquitously expressed in all tissues, and the shorter myosin-18Aβ is detected in hematopoietic cells (Mori et al., 2003), whereas the longer myosin-18Aγ has been found to be enriched in striated muscles (Horsthemke et al., 2019). Unlike the myosin-18Aα or myosin-18Aβ, the expression pattern of myosin-18B resembles that of myosin-18Aγ, which is highly enriched in cardiac and skeletal muscles and is detected at low level in other tissues (Salamon et al., 2003).

### Biochemical and Cellular Functions of Myosin-18s

#### Myosin-18A

**FIGURE 2:**
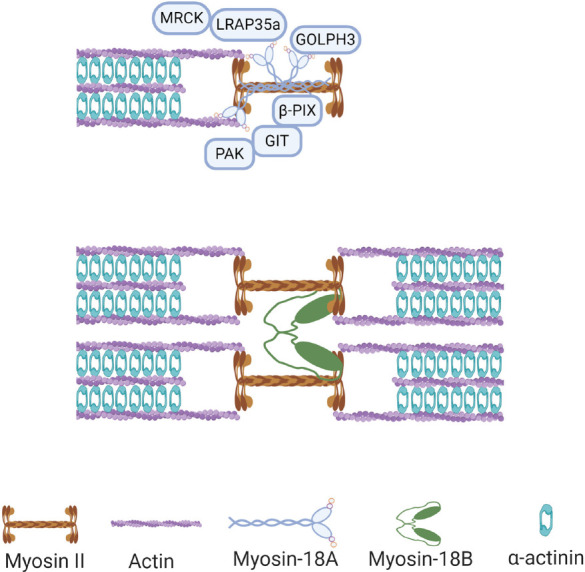
Functions of myosin-18s in non-muscle cells. Myosin-18A binds to actin filaments and bundled with myosin II in non-muscle cells, and it can interact with multiple proteins in this context, including MRCK, GOLPH3, and PAK2/βPIX/GIT1 complex. Myosin-18B assembles with myosin II filaments and facilitates fusion of adjacent myosin stacks during actomyosin bundle maturation.

Like many other myosins, myosin-18A can bind to actin and plays important roles in different actin structures (Taft and Latham, 2020). In 2005, Isogawa and colleagues used GFP-tagged fragments of human myosin-18A to test their actin-binding activity, and it was found that the N-terminal domain has a strong interaction with actin, which does not involve ATPase activity (Isogawa et al., 2005). Mori and colleagues reached similar conclusions at the same year with coimmunoprecipitation analysis showing that myosin-18A can self-associate through its coiled-coil domain and interact with actin under the mediation of the KE-rich domain (Mori et al., 2005). Later studies showed that both N-terminal and the motor domain of myosin-18A possess actin-binding activity (Guzik-Lendrum et al., 2013; Taft et al., 2013). However, because of its lack of motor activity, the molecular and cellular function of the interaction between myosin-18A and actin could not be the conventional actomyosin contraction, thus attracting attention of researchers on its specific roles on cytoskeleton. In a search for regulators of actomyosin retrograde flow essential for cell motility, Tan and colleagues found that myosin-18A interacted with Rac/Cdc42-binding kinase MRCK, a Rho GTPase effector kinase crucial for actomyosin retrograde flow (Tan et al., 2008). This interaction was found to be facilitated by the adaptor protein LRAP35a, and the tripartite complex formed by MRCK/myosin-18A/LRAP35a was responsible for the assembly of lamellar actomyosin bundles and of a subnuclear actomyosin network (Tan et al., 2008; Figure 2). The association between myosin-18A and lamellipodia and lamella was further demonstrated by the interaction between myosin-18A and PAK2/βPIX/GIT1 (p21-activated kinase2/PAK-interacting exchange factor-β/G protein–coupled receptor kinase interactor-1) complex, which is localized at lamellipodia and membrane ruffles (Hsu et al., 2010, 2014; Figure 2). Both MRCK and PAK2 are downstream effectors of small GTPase; however, the role of small GTPases in regulating the myosin-18A/PAK2/βPIX/GIT1 complex formation and function is still a puzzle. Furthermore, myosin-18A can coassemble with NM2 filaments and regulate the assembly of actomyosin bundles and stress fibers (Billington et al., 2015). Knocking down myosin-18A in prostate cancer cells increased circumferential NM2-associated actin filament arrays in the lamella (Makowska et al., 2015), different from phenotypes of knockdown of other myosins, suggesting its role in actomyosin is different from other myosins.

As mentioned above, myosin-18A can bind to F-actin, and it was found that this interaction can be further enhanced by binding of GOLPH3, a phosphoprotein of the Golgi membrane (Taft et al., 2013; Figure 2). GOLPH3 is known for its Golgi localization through interaction with phosphatidylinositol-4-phosphate (PI4P), a phospholipid enriched in Golgi membranes (Dippold et al., 2009). Moreover, in search for binding partners of GOLPH3, myosin-18A was identified to be interacting with GOLPH3 with its N-terminal extension and motor domains (Dippold et al., 2009). Recently, Rahajeng et al. (2019) demonstrate that GOLPH3 can drive PI4P-dependent membrane curvature of the Golgi. In this study, overexpression of amino-terminally tagged GOLPH3 unable to interact with myosin-18A results in both excessive tubulation of the Golgi and ineffective trafficking (Rahajeng et al., 2019). In addition, knockdown of myosin-18A showed similar phenotype, suggesting both GOLPH3-induced Golgi membrane curvature and recruiting of myosin-18A are required for forward trafficking from the Golgi to the plasma membrane (Rahajeng et al., 2019).

Except for being a GOLPH3 binding partner, myosin-18A was also identified as a receptor for lung surfactant protein A (SP-A). Yang et al. demonstrated that myosin-18A physically interacts with SP-A and proposed that a cryptic transmembrane domain in myosin-18A is responsible for the extracellular localization of its motor and C-terminal domains (Yang et al., 2005). Additionally, the interaction between myosin-18A and SP-A was also found to be important for macrophage activation (Yang et al., 2015). Besides, CD245, a highly conserved motor enzyme reported as a receptor for SP-A, has also been identified as myosin-18A (De Masson et al., 2016). Certainly, these studies shed light on the new feature of myosin-18A function. However, the obvious question raised is that how a protein that is generally observed to be cytosolic without transmembrane domain in most studies (9, 10) could act as a receptor for molecules found in the extracellular space. To understand this intriguing role of myosin-18A, additional studies will be needed.

#### Myosin-18B

Although myosin-18B is highly enriched in striated muscle cells, it is also present in non-muscle cells at low levels (Salamon et al., 2003). In the initial characterization, myosin-18B protein was found to have a special expression pattern during muscle differentiation: it is totally cytoplasmic in undifferentiated myoblast cells, but a fraction of this protein will translocate into the nucleus in differentiated muscle cells (Salamon et al., 2003). In addition, myosin-18B was found to be located on the Z-lines of striated muscle myofibrils, despite that conventional myosin is located in the A-bands and acts as a molecular motor for muscle contraction (Salamon et al., 2003; Ajima et al., 2008). However, recent studies have observed a different, if not totally opposite, localization of myosin-18B in muscular sarcomere, which appears to be localized to the A-bands, similar to the conventional myosins (Berger et al., 2017; Latham et al., 2020). Moreover, its localization during human embryonic stem cells (hESCs) to cardiomyocytes was also found to be opposite to the initial finding, which showed they localize in the nucleus of hESCs and become sarcomeric during cardiomyocyte differentiation (Latham et al., 2020). These obvious discrepancies may be due to different methods of detection, as multiple antibodies and GFP labels were used to examine the expression of myosin-18B, and some of these methods may not accurately reflect the endogenous expression pattern of myosin-18B. In non-muscle cells, myosin-18B has been reported to be expressed in punctate pattern throughout the cytoplasm, in membrane protrusions, and within stress fibers (Inoue et al., 2006; Ajima et al., 2008; Jiu et al., 2019).

Although the cellular localization of myosin-18B is not clear, some of its cellular functions have recently been discovered. In non-muscle cells, myosin-18B was found to promote the assembly of myosin II stacks, which are important for a variety of vital processes in cells (Jiu et al., 2019). Specifically, myosin-18B assembles with NM2 filaments and facilitates fusion of adjacent myosin stacks, which in turn promotes actomyosin bundle maturation (Jiu et al., 2019; Figure 2). In addition, *MYO18B* gene knockout cells display thin stress fibers, which can be rescued by AMPK activation, whereas myosin-18B overexpression leads to strong actin network, which can be abolished by CaMKK2 inhibition (Zhao et al., 2020), suggesting myosin-18B plays an important role in the actin stress fibers of the mechanically sensitive CaMKK2-AMPK-VASP signaling cascade (Zhao et al., 2020). In striated muscle cells, recent studies found that myosin-18B is an essential sarcomeric accessory protein, which can bind actin thin filaments in the forming sarcomere and be incorporated in the thick filaments, coinciding with striation onset of cardiomyocyte differentiation (Latham et al., 2020). Therefore, it was suggested that myosin-18B regulates higher-order organization of the cardiac sarcomere from within the thick filament (Latham et al., 2020).

### Myosin-18s in Muscle Development and Physiology

Because of the enriched expression of myosin-18B in cardiac and skeletal muscle, its role in striated muscle cells has been of particular interest to researchers. Its expression was found to be elevated during differentiation of C2C12 myoblast to myotubes (Salamon et al., 2003), and robust expression of myosin-18B was also observed as early as E9.5 mouse heart and E13.5 skeletal muscle precursors (Ajima et al., 2008), suggesting it has an important role in muscle development. Indeed, *MYO18B* gene knockout mice showed early embryonic lethality at E10.5 with severe cardiac defects (Ajima et al., 2008), suggesting cardiomyocyte development and function require myosin-18B. Electron microscopy analysis uncovered disrupted sarcomere structure in *MYO18B* knockout cardiomyocytes with defective alignment of thick and thin myofibril filaments (Ajima et al., 2008), indicating that myosin-18B functions in the process of myofibril organization. Two independent *MYO18B* mutants in zebrafish revealed additional role of myosin-18B in skeletal muscle integrity (Berger et al., 2017; Gurung et al., 2017). Zebrafish development can proceed to some extent without proper heart function, allowing examination of skeletal muscle defect of zebrafish *MYO18B* mutants with cardiac defects similar to the mouse mutant (Gurung et al., 2017). It was evident that sarcomeres of skeletal muscle were disorganized, and force could not be generated from the fast-twitch muscles of *MYO18B* mutant zebrafish (Berger et al., 2017; Gurung et al., 2017). Two independent findings of *MYO18B* mutations linked in human myopathies further confirmed the conserved function of myosin-18B in striated muscle development (Alazami et al., 2015; Malfatti et al., 2015). A homozygous missense mutation (c.6496G > T, p.Glu2166^∗^), which produced a truncated myosin-18B missing parts of the C-terminal domain, was found in patients with nemaline myopathy (Malfatti et al., 2015), characterized by dysmorphism, clinodactyly, hypotonia, muscle weakness, and cardiomyopathy. Another homozygous mutation (c.6905C > A) was identified in two patients with Klippel–Feil anomaly, presenting myopathy and distinct facial features (Alazami et al., 2015). This mutation results in a premature stop codon and induces nonsense-mediated decay of the mutant *MYO18B* mRNA, leading to a null phenotype. Interestingly, similar to the sarcomere defects in mouse and zebrafish *MYO18B* mutants, a failed assembly of mature sarcomere in striated muscles is found in all these patients, confirming myosin-18B functions in sarcomere assembly across different species. In addition, research associating *MYO18B* mutation with infant death accompanied by muscular defect was also reported recently (Armes et al., 2018). Possibly, the underlying mechanism is similar to the other reports, that myosin-18B is required for muscle development and function.

**FIGURE 3:**
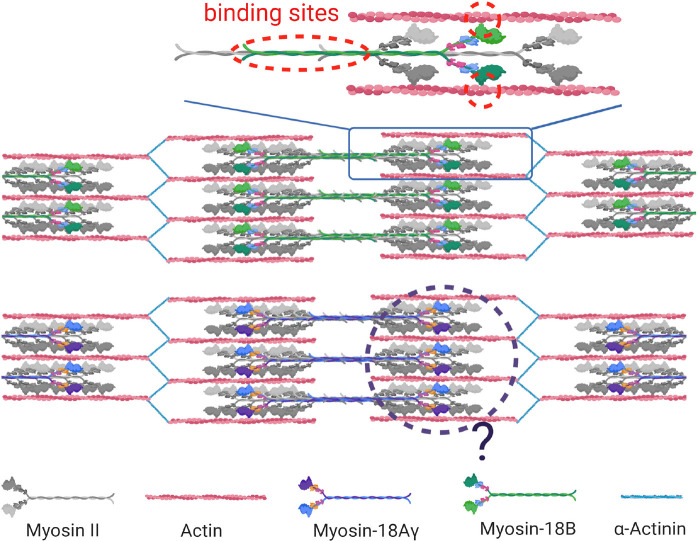
Function of myosin-18s in striated muscle cells. Myosin-18B and myosin-18 Aγ are localized in the sarcomere in striated muscle cells. Myosin-18B binds actin thin filaments and is incorporated in the thick myosin filaments *via* its coiled-coiled domain in sarcomeres of cardiomyocytes (molecular binding highlighted in red dotted circle). Whether mψoσιν-18Aγ possesses a similar function is yet to be discovered (black dotted circle).

Altogether, these similar loss-of-function phenotypes of myosin-18B in different organisms highlight the importance of myosin-18B in sarcomere assembly and striated muscle development. However, the phenotypic description did not provide sufficient evidence illuminating how myosin-18B regulates sarcomere assembly, given that it does not possess a motor activity, which is crucial to sarcomere function. It is possible that myosin-18B serves as a structural glue that ties newly synthesized actomyosin bundles into well-organized sarcomeric structures, similar to its function in facilitating stress fiber assembly in non-muscle cells (Jiu et al., 2019). A recent study in the differentiation from hESCs to cardiomyocytes revealed that myosin-18B binds actin thin filaments and is incorporated in the thick filaments during the onset of striation (Latham et al., 2020; Figure 3). Loss-of-function studies are warranted to further illuminate the function of human myosin-18B in cardiomyocytes.

Myosin-18A was thought to be absent in the muscle; thus, its involvement in striated muscle was revealed later than myosin-18B. In 2013, Bonn and colleagues found the sole myosin-18 in *Drosophila* is localized to the fusion pore between fusing myoblasts and around the Z-line of mature muscle cells (Bonn et al., 2013). However, the deficiency of myosin-18 did not affect muscle development because of possible compensation of other myosins (Bonn et al., 2013). Because it is the only myosin-18 in *Drosophila*, this observation did not provide a clear clue whether myosin-18A is functioning in muscle in vertebrates. There were two consecutive studies in zebrafish that revealed the role of myosin-18A in striated muscle development and function (Cao et al., 2014, 2016). Two myosin-18A genes *MYO18Aα* and *MYO18Aβ* exist in zebrafish, and both of them were expressed in somites during muscle development, and knockdown of *MYO18A*, as well as overexpression of the PDZ domain disrupted myofiber integrity (Cao et al., 2014). Subsequently, a few binding partners of myosin-18A in zebrafish was identified, including p190Rho-guanine nucleotide exchange factor (p190RhoGEF) and Golgin45, and their interaction was suggested to be required for extracellular matrix adhesion, Golgi apparatus formation, F-actin bundle organization, and eventually muscle integrity (Cao et al., 2016). A recently study in mouse confirmed that myosin-18A is essential for cardiac development and sarcomere organization (Horsthemke et al., 2019), as global knockout and cardiomyocyte-specific conditional knockout of *MYO18A* cause early embryonic lethality due to cardiac defect accompanied with sarcomere disruption in cardiomyocytes (Horsthemke et al., 2019). It also revealed that there is actually a cardiomyocyte-specific isoform of myosin-18A, myosin-18Aγ, which plays a key role in cardiac development (Horsthemke et al., 2019). Characterization of myosin-18Aγ showed that it has a long N-terminal domain containing a short PPII helix (Figure 1) and is localized within the A-band of sarcomeres (Horsthemke et al., 2019; Figure 3). However, the molecular function of myosin-18Aγ is largely unknown (Figure 3).

Knockout of either *MYO18A* and *MYO18B* in mouse led to embryonic lethality around E13.5 with similar sarcomere defects in cardiomyocytes, suggesting that they both may have essential role in sarcomere assembly with little overlapping functions or compensation. Currently, plenty *in vivo* evidence suggested that myosin-18s are important in cytoskeletal development of cardiomyocytes (Ajima et al., 2008; Berger et al., 2017; Gurung et al., 2017; Horsthemke et al., 2019), mainly through phenotypical description of loss-of-function mutants. However, *in vitro* or cellular functional studies are largely missing in cardiac or striated muscles, with the subcellular localizations of myosin-18s remaining unclear (Salamon et al., 2003; Latham et al., 2020). Therefore, extensive study on the molecular mechanisms of myosin-18s in sarcomere formation and maintenance is guaranteed.

### Myosin-18s in Cancer and Other Diseases

Both myosin-18A and myosin-18B have been implicated in cancers (Taft and Latham, 2020). Interestingly, apart from gene fusion and translocation events that link myosin-18s with cancer, their expression levels seem to be positively or negatively associated with different types of cancers, suggesting that their roles in cancer is also context dependent.

The first line of evidence showing the involvement of myosin-18s in cancer came from a report that *MYO18B* was found to be frequently deleted, mutated, and hypermethylated in lung cancers (Nishioka et al., 2002). And overexpression of *MYO18B* suppressed proliferation and anchorage-independent growth of lung cancer cells (Nishioka et al., 2002), suggesting myosin-18B serves as a tumor suppressor. Mutations or silenced expression of *MYO18B* was subsequently found in ovarian cancers (Yanaihara et al., 2004), colorectal cancers (Nakano et al., 2005), melanoma and pancreatic ductal adenocarcinoma (Bleeker et al., 2009), and neuroendocrine cancer (Bhatla et al., 2016). And in line with the initial discovery that *MYO18B* is silenced by hypermethylation of its promoter in lung cancers (Nishioka et al., 2002), hypermethylation of *MYO18B* promoter was found to be associated with weaker response to chemotherapies in ovarian cancers (Tomar et al., 2017), whereas hypomethylation of its promoter is associated with high *MYO18B* expression and favorable outcomes of T-cell acute leukemia cases (Haider et al., 2019). Although most studies on *MYO18B* in cancer support its role as a tumor suppressor, one report in hepatocellular carcinoma found that high *MYO18B* expression was associated with worse survival of this type of cancer, and knocking down *MYO18B* reduced proliferation and migration (Zhang et al., 2018).

Unlike the findings in *MYO18B*, *MYO18A* was mostly associated with cancers through gene fusion events, with *MYO18A* frequently found to be fused with other oncogenes such as FGFR1 (Walz et al., 2005), PDGFRB (Walz et al., 2009), and MLL (Ussowicz et al., 2012). In terms of its own function in cancer, it has been found that myosin-18A expression was increased in prostate cancer cells (Makowska et al., 2015), and its knockdown reorganized long NM2A-rich stress fibers and affected the cells’ ability to migrate (Makowska et al., 2015), suggesting a role of myosin-18A in prostate cancer metastasis (Peckham, 2016).

Other than myopathies and cancer, myosin-18s have also been implicated in other diseases. Myosin-18A was found to be localized to plasma membrane in human fibroblasts and translocate to viral assembly complex after human cytomegalovirus (HCMV) infection and plays a role in virus production (Jean Beltran et al., 2016). It was proposed that myosin-18A facilitates connections between vesicles loaded with virus and myosin filament containing NM2-binding domains (Billington et al., 2015), thus regulating the movement of the virus-loaded vesicles (Jean Beltran et al., 2016). In line with this study, myosin-18A was found to be required for the hepatitis C virus secretion (Bishe et al., 2012), possibly through regulating Golgi budding process *via* interaction with GOLPH3 (Dippold et al., 2009; Bishe et al., 2012). Myosin-18B was reported to be associated with schizophrenia (Takata et al., 2013) and mathematical ability (Ludwig et al., 2013), as single-nucleotide polymorphisms in myosin-18B were found to be linked to depth of intraparietal sulcus in the brain, which is responsible for mathematical abilities (Ludwig et al., 2013). However, the latter association was not supported by subsequent studies (Pettigrew et al., 2015).

## Conclusion and Perspectives

At the beginning of the 21st century, myosin-18A and myosin-18B were identified respectively, which together form the myosin-18 family. Unlike the conventional myosin family, myosin-18s lacks active ATPase-driven motor activity and is characterized by the presence of large N- and C-terminal extensions flanking a generic myosin core structure (Figure 1).

Myosin-18A plays multiple roles in different actin structures, including focal adhesions, actin stress fibers, lamellar actomyosin bundles, and Golgi apparatus (Figure 2). However, which splice isoform of myosin-18A localizes to Golgi or plays a role in Golgi morphology remains unknown. A recent study observed neither the colocalization nor any impact of reduced myosin-18Aα levels on Golgi morphology by immunofluorescence assay (Bruun et al., 2017). It was considered an unknown splice variant of myosin-18A, which may take part in this function, and further studies are needed to verify the speculation.

At present, three myosin-18A splice isoforms have been identified, namely, myosin-18Aα, myosin-18Aβ, and myosin-18Aγ (Horsthemke et al., 2019). Myosin-18Aα contains two distinct functional regions: a KE motif and a PDZ domain in its N-terminal extension, whereas myosin-18Aγ has a single PPII helix in its N-terminal extension (Figure 1). Myosin-18Aβ completely lacks these domains with a short N-terminal extension (Figure 1). Several studies showed a strong ATP-independent interaction of myosin-18A N-terminal extension with actin (Isogawa et al., 2005; Mori et al., 2005; Billington et al., 2015). But the precise structural and functional properties of the N-terminus, for example, the role of PPII, remain to be resolved. As for C-terminal extension, it is the least characterized domain of myosin-18A. So far, the Rho GTPase activator βPIX (PAK-interacting exchange factor-β) is the only one that was identified as a direct binding partner of the C-terminal domain (Hsu et al., 2010). Besides the common functions as a myosin family member, myosin-18A has a unique feature of binding to SPA at the cell surface, as SPA-receptor 210 (Yang et al., 2005). But myosin-18A has no predicted transmembrane domains. How is myosin18A transported to the cell membrane? Does the PDZ domain, which mediates membrane association, participate in this function? Further research may shed light on this unique role of myosin-18A.

The second XVIII myosin family member, myosin-18B, expresses highly in cardiac and skeletal striated muscles and is also widely distributed at a low level in organs and tissues. Except for the generic myosin configuration, myosin-18B comprises several unique domains, such as ERM domain in the tail region and a putative NLS in the C-term extension (Salamon et al., 2003; Figure 1). And the functional capabilities of these proposed domains remain to be assessed. Recently, several researches on the cellular localization and biochemical function of myosin-18B shed light on the understanding of this unique protein. Jiu et al. observed that myosin-18B plays as a “glue” molecule for assembling myosin II stacks and promotes the maturation of contractile actomyosin bundles (Jiu et al., 2019; Figure 2). Moreover, a follow-up study found myosin-18B also plays a critical role in the mechanosensitive regulation *via* CaMKK2-AMPK-VASP pathway (Zhao et al., 2020). Another controversial aspect of myosin-18B is its localization in striated muscle cells. While most conventional myosins are localized to the A-bands, myosin-18B was reported to be localized to the Z-lines (Salamon et al., 2003; Ajima et al., 2008), as well as A-bands (Berger et al., 2017; Latham et al., 2020), of striated muscle myofibrils. More precise and well-defined methods will be needed to explain the differences and confirm the localization of myosin-18B in sarcomere, which is critical to its function in muscle cells.

Myosin-18A and myosin-18B share ∼40% identity at the protein level and display unique roles at specific subcellular localities. The common localization myosin-18A and myosin-18B, such as stress fibers in non-muscle cells and sarcomeric structures in striated muscle, raises the question of whether they have distinct or overlapping functions within these shared sites. Both *MYO18A* and *MYO18B* knockout mice have sarcomere defects in cardiomyocytes and early embryonic lethality (Ajima et al., 2008; Horsthemke et al., 2019), suggesting that they have essential role in cardiac sarcomere assembly, whereas only MYO18B has been indicated in human congenital cardiac defects (Alazami et al., 2015; Malfatti et al., 2015). The detailed molecular function of myosin-18s in sarcomeres and whether they have distinct functions in cardiomyocytes require further research.

In terms of disease, both myosin-18A and myosin-18B have predominantly been investigated in cancers, including lung, prostate, and ovarian cancers (Nishioka et al., 2002; Yanaihara et al., 2004; Nakano et al., 2005; Walz et al., 2005, 2009; Bleeker et al., 2009; Ussowicz et al., 2012; Bhatla et al., 2016; Tomar et al., 2017; Zhang et al., 2018; Haider et al., 2019). Myosin-18A also participates in HCMV infection (Jean Beltran et al., 2016) and hepatitis C virus (HCV) secretion (Bishe et al., 2012). However, the molecular mechanisms of myosin-18A’s involvement in cancer and virus transport are still not known. Moreover, *MYO18B* gene, identified as a tumor suppressor, recently was reported as a tumor promoter in hepatocellular carcinoma progression (Zhang et al., 2018).

From all above, although functions of myosin-18 family at the molecular and cellular level have been greatly resolved during the recent 20 years, it is not hard to see that there is still a long way to go before completely unraveling the mysteries of members of this new branch within the myosin family.

## Author Contributions

YJ and KW initiated and made the outline of the manuscript. ZO, SZ, and YS drafted the manuscript with contributions from all other authors.

## Conflict of Interest

The authors declare that the research was conducted in the absence of any commercial or financial relationships that could be construed as a potential conflict of interest.
